# DPP4 Inhibitor Attenuates Severe Acute Pancreatitis-Associated Intestinal Inflammation via Nrf2 Signaling

**DOI:** 10.1155/2019/6181754

**Published:** 2019-11-15

**Authors:** Xiang Zhou, Weiming Wang, Cheng Wang, Chenlei Zheng, Xiangxiang Xu, Xiaofeng Ni, Shanshan Hu, Binbin Cai, Linxiao Sun, Keqing Shi, Bicheng Chen, Mengtao Zhou, Gang Chen

**Affiliations:** ^1^Department of Surgery, The First Affiliated Hospital of Wenzhou Medical University, Wenzhou, 325000 Zhejiang Province, China; ^2^Key Laboratory of Diagnosis and Treatment of Severe Hepato-Pancreatic Diseases of Zhejiang Province, The First Affiliated Hospital of Wenzhou Medical University, Wenzhou, 325000 Zhejiang Province, China

## Abstract

Severe acute pancreatitis (SAP) is a challenging disease with high morbidity and mortality, often complicated by multiple organ dysfunction syndrome (MODS). The intestine, a major organ involved in MODS, correlates strongly with the evolution of the disease. In this study, we demonstrated that the DPP4 inhibitor, sitagliptin, protects SAP-associated intestinal injury both in vitro and in vivo. These beneficial effects were achieved by suppressing oxidative stress and inflammatory responses. Moreover, in sitagliptin-treated SAP mice, expression of Nrf2 was induced and that of NF-*κ*B was reduced, compared to the control SAP mice. In addition, we used Nrf2^−/−^ mice to test the protective effect of Nrf2 during sitagliptin treatment of SAP; our results indicated that Nrf2^−/−^ mice had greater pancreatic and intestinal injury than wild-type mice. Taken together, high levels of ROS induced by SAP may be inhibited by sitagliptin, possibly by inactivating the Nrf2-NF-*κ*B pathway.

## 1. Introduction

The incidence of acute pancreatitis (AP) has been on the rise, with annual costs exceeding two billion dollars in the USA alone, inducing substantial medical and social burden [1]. Over 20% of AP cases is severe, with high morbidity and mortality rates [2]. In severe AP, multiple organ dysfunction syndrome (MODS) ensues following pancreatic necrosis [3]. The intestine, a major organ involved in MODS, is extremely vulnerable to inflammatory factors and correlates strongly with the evolution of the disease [4, 5]. Enterocyte damage and increased intestinal permeability induced by intestinal barrier dysfunction, making the bacterial translocation from the intestinal tract to the blood stream and/or distant organs, are believed to precede the evolution [6].

The gastrointestinal tract is a key source of reactive oxygen species (ROS). Despite the epithelial layer-provided protective barrier, ingested materials and pathogens can activate the epithelium, macrophages, and polymorphonuclear neutrophils to produce inflammatory cytokines and other mediators that contribute further to oxidative stress. Excessive ROS production in the inflamed mucosa may directly lead to intestinal barrier dysfunction in severe acute pancreatitis (SAP) rats [7]. Therefore, to seek effective treatment measures, early protection of the intestinal barrier function by reducing ROS production may be promising for the prevention and treatment of SAP.

Dipeptidyl peptidases (DPPs) belong to a family of proline-specific serine proteases capable of cleaving off a dipeptide from the amino-terminus. DPP4 is the best characterized peptidase in the family. DPP4 expression in the intestine is the highest among that of all organs, speculated to be widely distributed in the intestinal tract and closely related to the occurrence and development of various intestinal diseases. Recently, DPP4 expression is reportedly upregulated in some inflammatory diseases such as inflammatory bowel disease, atherosclerosis, obesity, and multiple sclerosis, suggesting its involvement in the pathogenesis of inflammation [8–10]. In our previous experiments, DPP4 in the intestinal tract was demonstrated to be significantly increased in the SAP mouse model and could be inhibited by the DPP4 inhibitor (DPP4i) sitagliptin. Sitagliptin was the first clinically used DPP4i, with the approval from the US Food and Drug Administration, for the treatment of type 2 diabetes in 2006 [11]. Grazia et al. [12] demonstrated that sitagliptin treatment decreased the levels of endothelial NOS monomer, responsible for the generation of ROS, although the amount of NO-producing dimeric form increased; markers of oxidative and nitrosative stress also decreased. Another recent publication [13] showed that using incretin-related DPP4 for treating diabetes increases metastatic capacity by activating the antioxidant transcription factor Nrf2 to eventually reduce ROS levels. To the best of our knowledge, the effect of the DPP4i sitagliptin on SAP-related intestinal damage has not yet been examined.

In this study, we demonstrate that sitagliptin is capable of protecting SAP-related intestinal damage and reducing the levels of ROS. We hypothesize that reduced levels of ROS produced by sitagliptin inhibit intestinal epithelial cell inflammation, which may be achieved by inactivating the Nrf2 pathway.

## 2. Materials and Methods

### 2.1. Materials

Dulbecco's modified Eagle's medium (DMEM), fetal bovine serum (FBS), and trypsin were purchased from Gibco (Grand Island, NY). Sitagliptin (sit; cat. No. HY-13749) was purchased from MedChemExpress. Lipopolysaccharide (LPS; from *E. coli* O127 : B8) was from Sigma. Cerulein (HY-A0190) was from MedChemExpress. Antibodies to DPP4 (ab187048), IL-6 (ab6672), IL-1*β* (ab9722), and TNF-*α* (ab6671) were from Abcam Inc. (Cambridge, USA). Antibodies to GAPDH (5174S), Nrf2 (12721), and NF-*κ*B (8242) were from Cell Signaling Technology Inc. (Beverly, MA, USA).

### 2.2. Cell Culture

IEC6 cells were obtained from The Cell Bank of Type Culture Collection of Chinese Academy of Sciences (Shanghai, China) and were cultured in DMEM supplemented with 10% FBS (Gibco, USA), 100 U/ml streptomycin, and 100 *μ*g/ml penicillin. Cells were maintained at 37°C and 5% CO_2_. Experiments were performed when the IEC6 cells reached 80%-90% confluence.

### 2.3. Detection of Cell Proliferation

Real-time quantitative proliferation was performed in E-plates 16, using the xCELLigence system (ACEA Biosciences, CA) and according to the manufacturer's instructions. Briefly, IEC6 cells (2 × 10^4^) were plated in serum-free medium in the plates. After 24 h, cells were treated with different concentrations of LPS (0, 1, 10, and 100 *μ*g/ml), with or without sitagliptin (100 *μ*M), and the cell index value was automatically monitored every 15 minutes.

### 2.4. Detection of Intracellular ROS

Intracellular ROS generation was determined by a Cellular Reactive Oxygen Species Detection Assay kit (ab186028; Abcam Inc., MA) using ROS Orange Dye as the molecular probe. IEC6 cells were planted in 6-well plates, after overnight, and then cultured in LPS (10 *μ*g/ml) medium with or without sit (100 *μ*M) for 30 min. Subsequently, cells were stained with ROS Orange Dye for 1 h in the dark, according to the protocol, and washed twice with phosphate buffered saline (PBS). The cells were fixed in 4% paraformaldehyde for 15 min. After three washes, the cells were blocked with 5% BSA in PBS for 30 min, and the nuclei were stained with DAPI solution. Pictures were taken by Leica TCS SP8 (Leica Microsystems, CMS GmbH, Wetzlar, Germany).

### 2.5. RNA Isolation and Quantitative Real-Time PCR

Total RNA was isolated from jejunum tissues and IEC6s using a TRIzol Kit (15596026; Invitrogen, Carlsbad, CA). To quantify the amount of mRNA, cDNA was synthesized from 1 *μ*g of total RNA in a final volume of 20 *μ*g by using the RevertAid First Strand cDNA Synthesis Kit (K1622; Thermo Fisher Scientific, MA). Next, quantitative real-time PCR (qRT-PCR) was performed using the SYBR-Green Master Mix kit (Takara Bio Inc., Japan) on a PCR Detection System (Bio-Rad, CFX96) using standard conditions, following the manufacturer's instructions. Results were analyzed using the 2^-*ΔΔ*Ct^ method, and *β*-actin was amplified as an internal standard. All the primer sequences are listed in Table 1.

### 2.6. Western Blot Analysis

IEC6 cells and C57BL/6 mice intestinal issue protein were extracted using RIPA buffer (P0013B; Beyotime Biotechnology, Shanghai, China), supplemented with phenylmethane-sulfonyl fluoride (ST506; Beyotime Biotechnology) and PhosSTOP (Roche). Protein concentrations were determined using the BCA assay kit (P0012; Beyotime Biotechnology). After denaturation, equal amounts of protein were separated by SDS-PAGE and transferred onto PVDF membranes (Millipore, Billerica, MA). After transferring, the membranes were blocked with 5% skim milk for 2 h at room temperature. Then, the membranes were probed with primary antibodies overnight at 4°C and thereafter incubated with the appropriate secondary antibodies for 1 h at room temperature. Protein bands were detected using Image Lab Software (Bio-Rad Laboratories Inc., Berkeley, CA).

### 2.7. Animal Experiment Protocol

Six-week-old C57BL/6 mice weighing approximately 20–25 g were purchased from Shanghai Slake Laboratory Animal Co. Ltd. (Shanghai, China). Nrf2^−/−^ mice on C57BL background were obtained from the Experiment Animal Center of Nanjing Medical University (Jiangsu, China). All animal experiments were following the guidelines set by the Ethical Committee of Wenzhou Medical University and approved by the Laboratory Animal Management Committee of Zhejiang Province. Cerulein pancreatitis was induced as previously described [14]. Briefly, mice were treated by 7 h intraperitoneal (IP) injections of a dose of cerulein (50 *μ*g/kg). The cerulein plus LPS model was induced by IP injection of lipopolysaccharide (10 mg/kg) immediately after the 7 h IP injections of cerulein (50 *μ*g/kg). Mice were killed 24 h after the last cerulein injection. We randomly divided the mice into four groups (*n* = 8/group): (a) the control group, (b) the SAP group, (c) the SAP+ sit (100 mg/kg, IP) group, and (d) the SAP + sit (200 mg/kg, IP) group. Sit (100 or 200 mg/kg, IP) was administered 1 h prior to the first IP injection of cerulein. All surgeries were performed under intraperitoneal ketamine (100 mg/kg) and xylazine (5 mg/kg).

### 2.8. Histopathological Analysis

To investigate the protective effects of sitagliptin on intestinal inflammation induced by SAP, pancreatic and small intestine tissues were collected, the samples were fixed in 4% paraformaldehyde solution for 1-3 days, embedded in paraffin, and cut into 4 mm thick sections, which were processed for hematoxylin and eosin (H&E) staining. The morphological changes were observed under a microscope by two pathologists in a blinded manner. An assessment of vacuolization, edema, acinar cell necrosis, and inflammatory cell infiltration was carried out. Pancreatic injury was scored on a scale of 0–3 according to four items: edema (0 absent, 1 focally increased between lobules, and 2 diffusely increased); inflammatory cell infiltrate (0 absent, 1 in ducts (around ductal margins), 2 in the parenchyma (<50% of the lobules), and 3 in the parenchyma (>50% of the lobules)); hemorrhage and fat necrosis (0 absent, 1 (1–2 foci), 2 (3–4 foci), and 3 (>5 foci)); and acinar necrosis (0 absent, 1 periductal necrosis (<5%), and 2 focal necrosis (5–20%), and 3 diffuse parenchymal necrosis (20–50%)), as previously described [15, 16]. The pathological changes in the intestinal tissues were observed under the light microscope, and the pathological injury of the intestinal tissues was scored according to the ParkScore [17, 18]: normal mucosa (grade 0); subepithelial vacuolization and small subepithelial space at villi tips (grade 1); presence of more extended subepithelial space (grade 2); epithelial lifting extended along villi sides (grade 3), denuded villi (grade 4), loss of villi (grade 5), crypt layer infarction (grade 6), transmucosal infarction (grade 7), and transmural infarction (grade 8).

### 2.9. CD26/DPP4 Activity Assay and ELISA of IL-6 and IL-1*β*

At the end point of the study, mice were anesthetized with intraperitoneal ketamine (100 mg/kg) and xylazine (5 mg/kg). We collected the blood in coagulation tubes and centrifuged (7000 × g, 10 min, 4°C) and stored the supernatants as serum samples. CD26/DPP4 activity in mouse serum was measured using the CD26/DPPIV Assay kit (Enzo Life Sciences, Farmingdale, NY) according to the manufacturer's protocol. Release of IL-6 and IL-1*β* in mouse serum was measured using ELISA kits (Abcam Inc., USA), according to the manufacturer's instructions. Absorbance at 450 nm was recorded using a microplate reader (Bio-Rad).

### 2.10. Detection of Malondialdehyde (MDA) Concentration and Superoxide Dismutase (SOD) Activity

The intestinal tissues were homogenized and centrifuged at 12000 × g for 15 min before collecting the supernatant for spectrophotometric investigation. Protein concentrations were determined using the BCA assay kit. The concentrations of MDA and activities of SOD were detected using the appropriate kits (Beyotime Biotech, Inc., Jiangsu, China) and according to the manufacturer's instructions.

### 2.11. Statistical Analyses

Values are presented as the mean ± standard deviation (SD). Statistical analysis was performed using GraphPad Prism 7.0 (GraphPad Software, San Diego, CA). One-way ANOVA was used to determine differences among more than two groups; Tukey's multiple comparisons test were used to compare the mean of every other column. The results were calculated using data from three independent experiments. *p* < 0.05 was considered statistically significant.

## 3. Results

### 3.1. Sitagliptin Protects LPS-Stimulated IEC6 Cells

**Figure 1:**
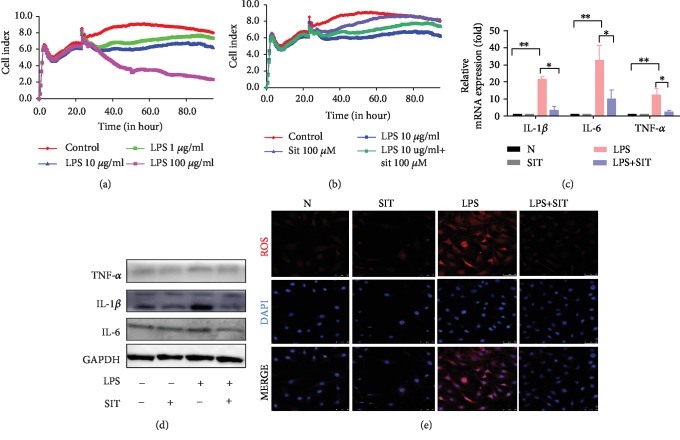
Sitagliptin protects IEC6 cells stimulated by LPS. (a) Cell viability was measured by RTCA cell proliferation detection exposure to various concentrations of LPS that range from 1 *μ*g/ml to 100 *μ*g/ml. (b) Cell proliferation detection by RTCA exposure to LPS 10 *μ*g/ml, plus sitagliptin (100 *μ*M) or none. (c) Real-time PCR results of IL-1*β*, IL-6, and TNF-*α* in IEC6 cells after incubating in LPS (10 *μ*g/ml) plus sitagliptin (100 *μ*M) or none for 24 h. (d) Western blotting results of IL-1*β*, IL-6, and TNF-*α* in IEC-6 cells after incubating in LPS (10 *μ*g/ml) plus sitagliptin (100 *μ*M) or none for 24 h. (e) The fluorescence intensity of ROS (red) after culturing with LPS (10 *μ*g/ml) media plus sitagliptin (100 *μ*M) or none for 30 min was detected using Leica TCS SP8 (×400). Cell nuclei were counterstained with DAPI (blue). Data are presented as mean ± standard error of the mean (SEM) (*n* = 3). ^∗∗^*p* < 0.05, compared with the N group. ^∗^*p* < 0.05, compared with the LPS group. N, the control group; SIT, the 100 *μ*M sitagliptin group; LPS, the 10 *μ*g/ml lipopolysaccharide group; LPS+SIT, the 10 *μ*g/ml lipopolysaccharide+100 *μ*M sitagliptin group.

CD26/DPP4 has been reported to regulate cell proliferation in several instances [19]. Therefore, cell proliferation assays were performed to determine the potential effect of DPP4 inhibition on IEC6 after LPS. RTCA for cell proliferation detection revealed that LPS causes a significant reduction in the proliferative capacity of the IEC6 cells in a concentration-dependent manner (*p* < 0.05) (Figure 1(a)). We chose LPS (10 *μ*g/ml) in the following experiments, whereas sitagliptin (100 *μ*M) suppressed the decrease in the cell significantly (*p* < 0.05) (Figure 1(b)). These results indicated the protective effects of sitagliptin on IEC6 after LPS stimulation. The influence of sitagliptin stimulation on LPS-induced IEC6 cells was detected by real-time PCR and Western blot. As shown in Figures 1(c) and 1(d), the expression of IL-1*β*, IL-6, and TNF-*α* decreased significantly (*p* < 0.05). We also demonstrated that LPS significantly increased the ROS levels, using the ROS Orange Dye to detect changes in intracellular ROS and analyzing with Leica TCS SP8. However, when IEC6 cells were preincubated with sitagliptin (100 *μ*M) for 1 h, ROS levels were obviously decreased (Figure 1(e)). Thus, the administration of sitagliptin markedly reduced the production of inflammatory factors and ROS, thereby attenuating injury.

### 3.2. Sitagliptin Attenuates SAP-Induced Intestinal Injury in Mice

**Figure 2:**
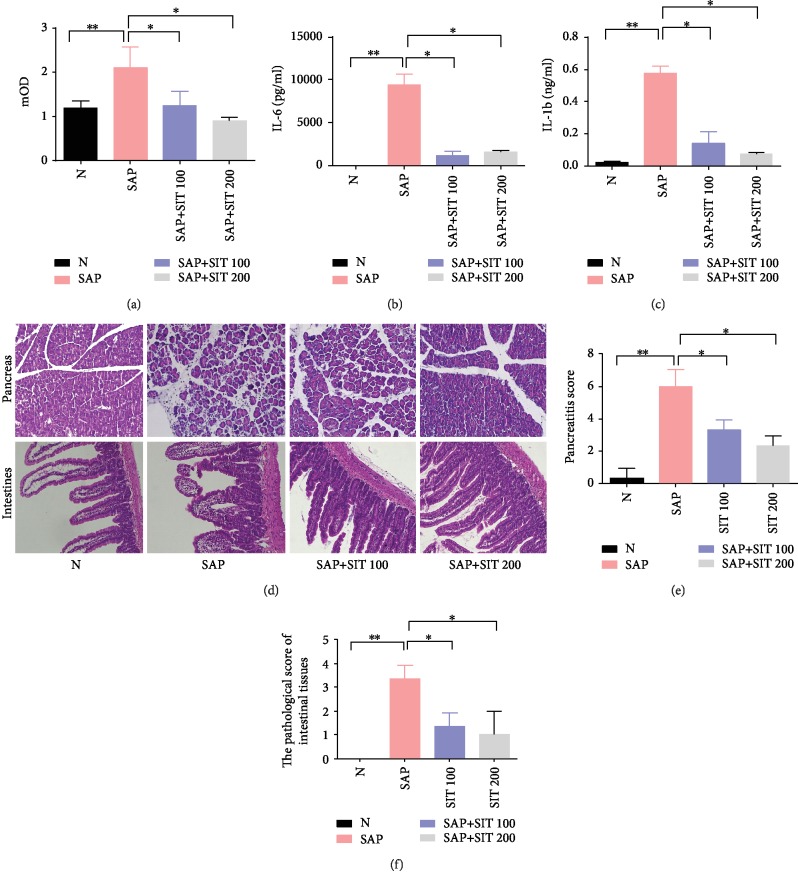
Sitagliptin attenuates SAP-induced intestine injury in mice. Mice received sitagliptin (100 or 200 mg/kg, IP) or vehicle 1 h prior to cerulein+ LPS (SAP model). The serum, pancreas, and intestine tissues were then harvested 24 h after the last intraperitoneal injection. (a) DPP4 activity in serum. (b) The cytokines' IL-6 in serum. (c) The cytokines' IL-1*β* in serum. (d) Representative hematoxylin and eosin- (H&E-) stained pancreas and intestine sections (×200). (e) The pancreatitis score. (f) Histological scores to evaluate the degree of injury. Slides were evaluated by two independent investigators in a blinded manner. Data are presented as mean ± standard error of the mean (SEM) (*n* = 3). ^∗∗^*p* < 0.05, compared with the N group. ^∗^*p* < 0.05, compared with the SAP group. N, the control group; SAP, the severe acute pancreatitis group; SAP+SIT 100, the severe acute pancreatitis+100 *μ*M sitagliptin group; SAP+SIT 200, the severe acute pancreatitis+200 *μ*M sitagliptin group.

First, to assess the systemic inhibitory effect of sitagliptin on DPP4 activity in mice, plasma DPP4 activity was measured. SAP was seen to significantly increase DPP4 activity while sitagliptin produced dose-dependent inhibition of DPP4 activity in the mouse model (Figure 2(a)). We next assessed whether sitagliptin has protective effects on SAP-induced intestinal injury in mice. As shown in Figures 2(b) and 2(c), SAP increased IL-6 and IL-1*β* levels in serum (*p* < 0.05) while sitagliptin (100 or 200 mg/kg) treatment inhibited the increase significantly (*p* < 0.05). The H&E staining assay showed that edema of acinar and inflammatory cell infiltrates in pancreas tissue and villi with large subepithelial spaces in the intestinal tissue was present in the SAP group but not in the control group, and there were little pathological features in the sitagliptin group compared to the SAP group (Figures 2(d)–2(f)). These results indicated that sitagliptin administration can attenuate injury to the pancreas and intestines.

### 3.3. Alteration of Oxidative Stress Responses and Nrf2-Mediated Signaling Pathway in Sitagliptin-Treated SAP Mice

**Figure 3:**
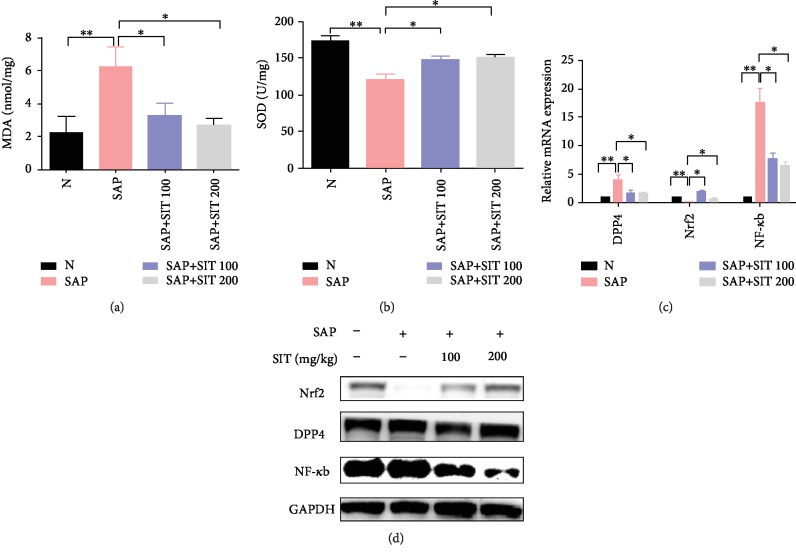
The alteration of oxidative stress responses and Nrf2-mediated signaling pathway in sitagliptin-treated SAP mice. The pancreas and intestine tissues were harvested 24 h after the last intraperitoneal injection. (a) Concentration of MDA in intestinal tissue from the different groups. (b) Activity of SOD in the intestinal tissue from the different groups. (c) Real-time PCR results of DPP4, Nrf2, and NF-*κ*B in different groups. (d) Western blotting results of DPP4, Nrf2, and NF-*κ*B in different groups. Data are presented as mean ± standard error of the mean (SEM) (*n* = 3). ^∗∗^*p* < 0.05, compared with the N group. ^∗^*p* < 0.05, compared with the SAP group. N, the control group; SAP, the severe acute pancreatitis group; SAP+SIT 100, the severe acute pancreatitis+100 *μ*M sitagliptin group; SAP+SIT 200, the severe acute pancreatitis+200 *μ*M sitagliptin group.

To investigate whether sitagliptin reduced ROS in the course of SAP in vivo, the oxidative stress-related indicators, malonyldialdehyde (MDA) and superoxide dismutase (SOD) activity, were measured in the intestinal tissues. Tissue concentrations of MDA were higher in the SAP group compared to those in the control group, while sitagliptin significantly decreased the MDA concentration caused by SAP (Figure 3(a)). Conversely, SOD activity was visibly less in the SAP group than in the control group, and antioxidant enzyme concentrations were higher in the sitagliptin group than in the SAP group (Figure 3(b)). Furthermore, sitagliptin increased the expression of Nrf2 and decreased the expression of DPP4, in contrast to that in SAP group (Figures 3(c) and 3(d)). In addition, the in vivo experiments showed no significant difference between 100 mg/kg and 200 mg/kg sitagliptin treatment groups. We speculated that the Nrf2/NF-*κ*B pathway may play a role in the protective effects of sitagliptin.

### 3.4. Anti-Inflammatory Effect of Sitagliptin Was Significantly Reduced in Nrf2^−/−^ Mice

**Figure 4:**
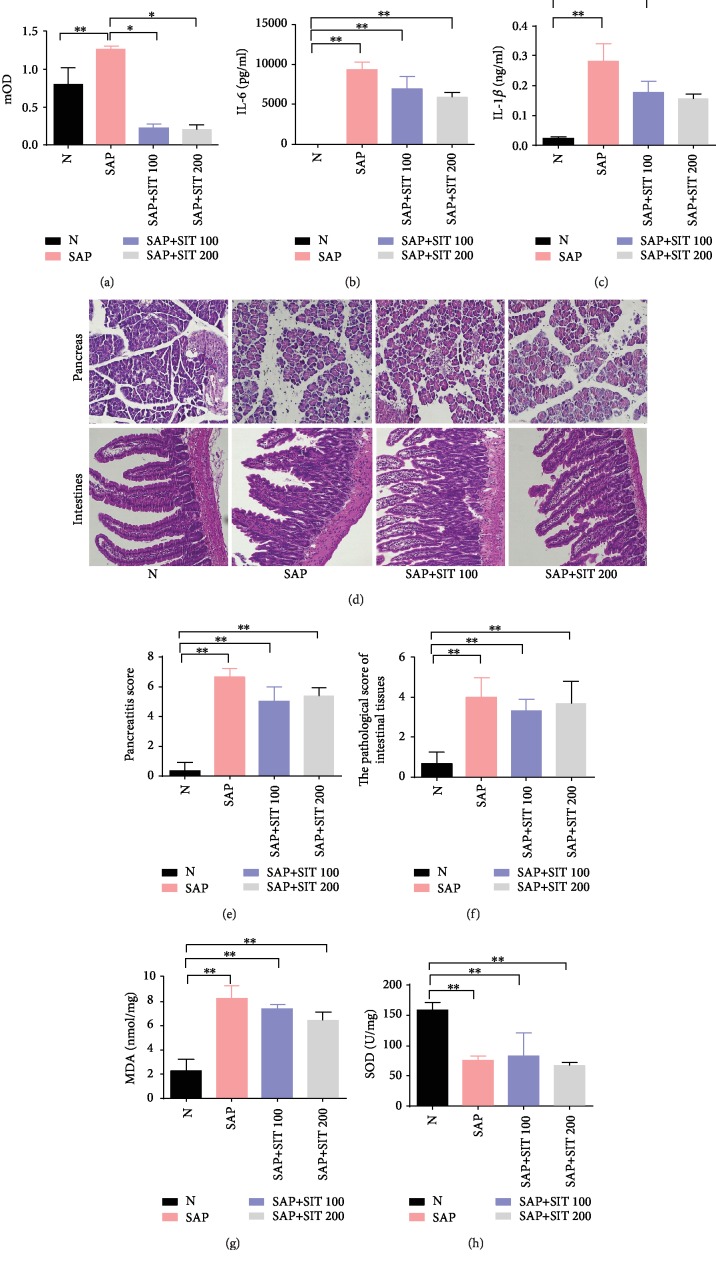
The anti-inflammatory effect of sitagliptin was significantly reduced in Nrf2^−/−^ mice. Nrf 2^−/−^ mice received sitagliptin (100 or 200 mg/kg, IP) or vehicle 1 h prior to cerulein+LPS (SAP model). The serum, pancreas, and intestine tissues were then harvested 24 h after the last intraperitoneal injection. (a) DPP4 activity in serum. (b) The cytokines' IL-6 in serum. (c) The cytokines' IL-1*β* in serum. (d) Representative hematoxylin and eosin- (H&E-) stained pancreas and intestine sections (×200). (e) The pancreatitis score. (f) Histological scores to evaluate the degree of injury. Slides were evaluated by two independent investigators in a blinded manner. (g) Concentration of MDA in intestinal tissues from the different groups. (h) Activity of SOD in intestinal tissues from the different groups. Data are presented as mean ± standard error of the mean (SEM) (*n* = 3). ^∗∗^*p* < 0.05, compared with the N group. ^∗^*p* < 0.05, compared with the SAP group. N, the control group; SAP, the severe acute pancreatitis group; SAP+SIT 100, the severe acute pancreatitis+100 *μ*M sitagliptin group; SAP+SIT 200, the severe acute pancreatitis+200 *μ*M sitagliptin group.

To investigate the impact of Nrf2 deletion on the anti-inflammatory properties of sitagliptin, we built the SAP model in Nrf2^−/−^ mice. 24 h after the last intraperitoneal injection, some mice died in the SAP groups with or without sitagliptin treatment after 24 h. At the same time, sitagliptin could effectively inhibit DPP4 activities (Figure 4(a)). This may be the reason for partially anti-inflammatory effects in Nrf2^−/−^ mice, although there was no significant difference in IL-6 and IL-1*β* concentrations between the treatment groups and the SAP group (Figures 4(b) and 4(c)). The concentrations of IL-6 and IL-1*β* significantly increased after the combined cerulein and LPS treatment compared to that in WT mice. Sitagliptin treatment also proved less effective in the Nrf2^−/−^ mice (Figures 4(b) and 4(c)). H&E staining assay confirmed that sitagliptin treatment could not reverse the edema and leukocyte infiltration trend in pancreas tissue, and the epitheliums damage at the tips of the villi in intestinal tissue observed in the Nrf2^−/−^ mice, compared to that in WT mice (Figures 4(g)–4(h)). Tissue concentrations of MDA were higher in the SAP group compared to that in the control group, while sitagliptin could not decrease the MDA concentration caused by SAP (Figure 4(g)). Conversely, SOD activity was significantly less in the SAP group than in the control group, and there was no significant difference between the sitagliptin groups and the SAP group (Figure 4(h)). Our results showed that the protective effect of sitagliptin on the intestine was abolished in Nrf2^−/−^ mice. Thus, the positive effect of sitagliptin on SAP-related intestine injury may depend on Nrf2 activation.

## 4. Discussion

SAP is a challenging disease with high morbidity and mortality and often complicated by multiple organ failure. Two phases of SAP have been observed clinically: the early toxicoenzymatic phase and the later septic phase [20]. Translocation of bacteria within the intestine, caused by intestine damage, is considered a second hit following SAP, leading to MODS. Therefore, it is necessary to find an effective therapy for the management of SAP-associated intestinal injury. Snarska et al. [21] had hypothesized that DPP4 gene polymorphism influences disease susceptibility and AP severity, which is widely distributed in the intestinal tract and speculated to be closely related to the occurrence and development of various intestinal diseases. It is highly relevant to target DPP4 substrate ligands, involved in a variety of major clinically acute and chronic injury/disease, including inflammation [22]. There is recent evidence that soluble DPP4 (sDPP4) enhances the transcription of IL-6 and TNF-*α* induced by LPS in THP-1 cells and monocytes [23], and sDPP4 itself can act as a proinflammatory signaling molecule in human smooth muscle cells via protease-activated receptor 2 [24]. In addition, DPP4i reduced atherosclerotic lesions in the aortas of apoE-deficient mice in association with downregulation of gene expression levels of the proinflammatory mediators IL-1*β*, TNF-*α*, and IL-6. It also suppressed proinflammatory cytokine release from human macrophages after stimulation by LPS at both gene and protein expression levels [25], thus suggesting that DPP4i has the potential to exert positive pleiotropic effects on inflammatory diseases. Consistent with these recent reports, our results also showed that sitagliptin can protect intestinal cell line, IEC6 cells, after LPS stimulation. Moreover, SAP significantly increased DPP4 activity while sitagliptin caused a dose-dependent inhibition of DPP4 activity and inhibition of the inflammatory response in this mouse model. Downregulation of the DPP4 activity could be an aspect of DPP4i inhibition of inflammation, but the results in Nrf2^−/−^ mice showed that this was not the main reason why DPP4i inhibits inflammation in intestinal tissues.

**Figure 5:**
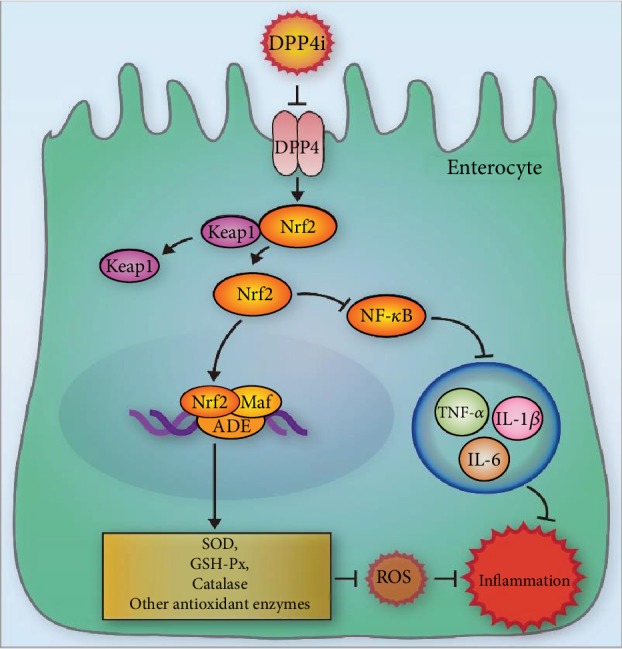
A model illustrating the potential molecular mechanism involved in the DPP4i-treated IEC6 cells.

ROS is closely related with the activation of inflammatory cascades and tissue damage in acute pancreatitis [26]. It is considered indispensable and a threat in the gastrointestinal tract, when host defense and redox signaling is involved [27]. Results from the present study showed that sitagliptin significantly altered the oxidant/antioxidant balance. Sitagliptin decreased ROS in the in vitro inflammation model and depressed the MDA level accompanied by increased SOD activity in the SAP mouse model. Increasing evidence confirmed that Nrf2 is closely related to ROS [28]. Nrf2 is a transcriptional activator that can serve as a sensor for oxidative stress. Nrf2 transcription factor modulates the expression of defensive genes coding detoxifying enzymes and antioxidant proteins. In response to attack by electrophiles or ROS, Nrf2 is switched on and off via distinct mechanisms [29]. In SAP, sitagliptin treatment was shown to increase the expression of Nrf2 in the intestines. Similarly, the concentrations of the inflammatory cytokines (IL-1*β* and IL-6) in the Nrf2^−/−^ mice was significantly increased than in WT mice and that the latter had a greater reduction in cytokines with sitagliptin than the Nrf2^−/−^ mice. Moreover, our results showed that the protective effect of sitagliptin on the intestine was abolished in Nrf2^−/−^ mice (Figure 4). We speculated that the main cause of sitagliptin inhibiting inflammation should be the Nrf2/NF-*κ*B pathway (Figure 5).

NF-*κ*B is closely involved with the inflammatory responses [30]. Studies using mouse models of ischemia/reperfusion injury [31] and neurodegenerative and cerebrovascular diseases [32] had suggested that NF-*κ*B associated with inflammatory response can be inhibited by regulating Nrf2 expression. As a result of oxidative stress, IKK*α* or IKK*β* phosphorylates IkBs leading to the release of NF-*κ*B, and its translocation into the nucleus. NF-*κ*B binds to DNA and upregulates the transcription of many inflammatory genes like cytokines, chemokines, and receptors of advanced glycation end products [33]. In the current study, sitagliptin pretreatment showed a remarkable inhibitory effect on the NF-*κ*B pathway after the activation of Nrf2, following significantly decreased IL-1*β* and IL-6 levels in mouse serum.

## 5. Conclusions

We showed that sitagliptin significantly protected intestinal epithelial cells by attenuating the oxidative stress and inflammatory response in vitro and in a mouse model of SAP. The Nrf2 expression was active in the process. And mice deficient in Nrf2 were more vulnerable to inflammatory response in SAP. Moreover, the inactivation of the NF-*κ*B pathway caused by the upregulated of Nrf2 also has a pivotal role on sitagliptin-reduced inflammatory response in the intestines (Figure 5). These findings may serve as the basis for the development of therapeutic strategies using sitagliptin that could assist in the recovery from SAP in clinical practice.

## Figures and Tables

**Table 1:** Sequences of the primers used for quantitative real-time PCR.

	Gene	Forward primer (5′–3′)	Reverse primer (5′–3′)
IEC-6	*β*-Actin	TGTCACCAACTGGGACGATA	GGGGTGTTGAAGGTCTCAAA
	IL-1*β*	ATCTCACAGCAGCATCTCGACAAG	CACACTAGCAGGTCGTCATCATCC
	IL-6	AGGAGTGGCTAAGGACCAAGACC	TGCCGAGTAGACCTCATAGTGACC
	TNF-*α*	GCATGATCCGAGATGTGGAACTGG	CGCCACGAGCAGGAATGAGAAG

Mouse	*β*-Actin	GTGCTATGTTGCTCTAGACTTCG	ATGCCACAGGATTCCATACC
	DPP4	CAGTGGCTCAGGAGGATTCAGAAC	TCAACATGCTGCTGCTCGGATG
	Nrf2	AGGACATGGAGCAAGTTTGG	TCTGTCAGTGTGGCTTCTGG
	NF-*κ*B	GTGGAGGCATGTTCGGTAGT	GACTCCGGGATGGAATGTAA

## Data Availability

The data used to support the findings of this study are available from the corresponding author upon request.

## Abbreviations

DPP4: Dipeptidyl peptidase-4
Nrf2: Nuclear factor erythroid factor 2
SAP: Severe acute pancreatitis
MODS: Multiple organ dysfunction syndrome
NF-*κ*B: Nuclear factor kappa B
ROS: Reactive oxygen species
AP: Acute pancreatitis
DPP4i: Dipeptidyl peptidase-4 inhibitor
NOS: Nitric oxide synthase
NO: Nitric oxide
DMEM: Dulbecco's modified Eagle's medium
FBS: Fetal bovine serum
LPS: Lipopolysaccharide
IEC6: Intestinal epithelial cell line
MDA: Malondialdehyde
SOD: Superoxide dismutase.
